# A tutorial on the case time series design for small-area analysis

**DOI:** 10.1186/s12874-022-01612-x

**Published:** 2022-04-30

**Authors:** Antonio Gasparrini

**Affiliations:** 1 Department of Public Health, Environments and Society, London School of Hygiene and Tropical Medicine (LSHTM), 15-17 Tavistock Place, London, WC1H 9SH UK; 2 Centre for Statistical Methodology, London School of Hygiene & Tropical Medicine (LSHTM), Keppel Street, London, WC1E 7HT UK

**Keywords:** Case time series, Small-area, Time series, Distributed lag models, Study design, Temperature

## Abstract

**Background:**

The increased availability of data on health outcomes and risk factors collected at fine geographical resolution is one of the main reasons for the rising popularity of epidemiological analyses conducted at small-area level. However, this rich data setting poses important methodological issues related to modelling complexities and computational demands, as well as the linkage and harmonisation of data collected at different geographical levels.

**Methods:**

This tutorial illustrated the extension of the case time series design, originally proposed for individual-level analyses on short-term associations with time-varying exposures, for applications using data aggregated over small geographical areas. The case time series design embeds the longitudinal structure of time series data within the self-matched framework of case-only methods, offering a flexible and highly adaptable analytical tool. The methodology is well suited for modelling complex temporal relationships, and it provides an efficient computational scheme for large datasets including longitudinal measurements collected at a fine geographical level.

**Results:**

The application of the case time series for small-area analyses is demonstrated using a real-data case study to assess the mortality risks associated with high temperature in the summers of 2006 and 2013 in London, UK. The example makes use of information on individual deaths, temperature, and socio-economic characteristics collected at different geographical levels. The tutorial describes the various steps of the analysis, namely the definition of the case time series structure and the linkage of the data, as well as the estimation of the risk associations and the assessment of vulnerability differences. R code and data are made available to fully reproduce the results and the graphical descriptions.

**Conclusions:**

The extension of the case time series for small-area analysis offers a valuable analytical tool that combines modelling flexibility and computational efficiency. The increasing availability of data collected at fine geographical scales provides opportunities for its application to address a wide range of epidemiological questions.

## Introduction

The field of epidemiology has experienced profound changes in the last decade, with the fast development of data science methods and technologies. Modern monitoring devices, for instance remote sensing instruments or mobile wearables [1], provide real-time measurements of a variety of risk factors with unparalleled coverage, quantity, and precision. Similarly, advancements in linkage procedures [2], together with improved computational capabilities, storage, and accessibility [3], offer epidemiologists rich and high-quality data to investigate health risks.

The availability of data on health outcomes and exposures with increased resolution is the main driver of the rising popularity of epidemiological analyses at small-area level [4]. Originally developed in spatial analysis, small-area methods have been then extended for spatio-temporal data to analyse observations collected longitudinally [5, 6]. Similarly to traditional studies based on aggregated data, these investigations often make use of administratively collected information, usually more available to researchers and less sensitive to confidentiality restrictions. Nonetheless, these studies provide a richer data framework, merging information gathered from various sources at multiple geographical levels. The aggregation of information at finer spatial scales makes small-area studies less prone to ecological fallacies affecting traditional investigations using large-scale aggregations, and the availability of more detailed data can inform about more complex epidemiological mechanisms. Still, this context poses non-trivial practical and methodological problems, for instance high computational requirements related to the size of the data, and modelling issues due to their complexity [7].

The *case time series* (CTS) design is a methodology recently proposed for epidemiological analyses of short-term risks associated with time-varying exposures [8]. The design combines the modelling flexibility of time series models with the self-matched structure of case-only methods [9], providing a suitable framework for complex longitudinal data. Originally illustrated in individual-level analyses, the CTS design can be easily adapted for studies using data aggregated over small areas. This extension makes available a flexible methodology applicable for a wide range of research topics.

In this contribution, we provide a tutorial on the application of the CTS design for the analysis of small-area data. The tutorial describes several steps, including data gathering and linkage, modelling of epidemiological associations, and definition of effect summaries and outputs. The associated with non-optimal temperature in London, United Kingdom. The example is fully reproducible, with data and code in the R software available in a GitHub repository.

## The case time series data structure

The real-data example is based on a dataset published by the Office of National Statistics (ONS), reporting the deaths that occurred in London in the summer period (June to August) of two years, 2006 and 2013. The data are aggregated by day of occurrence across 983 *middle layer super output areas* (MSOAs), small census-based aggregations with approximately 7,200 residents each. The dataset includes the death counts for both the age group 0–74 and 75 and older, which are combined in total numbers of daily deaths for this analysis. The paragraph below describes how these data must be formatted in a CTS structure.

The CTS design is based on the definition of *cases*, representing observational units for which data are longitudinally collected. The design involves the definition of case-specific series of continuous sequential observations. In the applications of the original article presenting the methodology [8], cases were represented by subjects, but the design can be extended by defining the observational units as small geographical areas. In this example, the process implies the aggregation of the mortality data in MSOA-specific daily series of mortality counts, including days with no death. It is worth noting that the design is similarly applicable with different types of health outcomes, for instance continuous variables obtained by averaging measurements within each area.

**Fig. 1:**
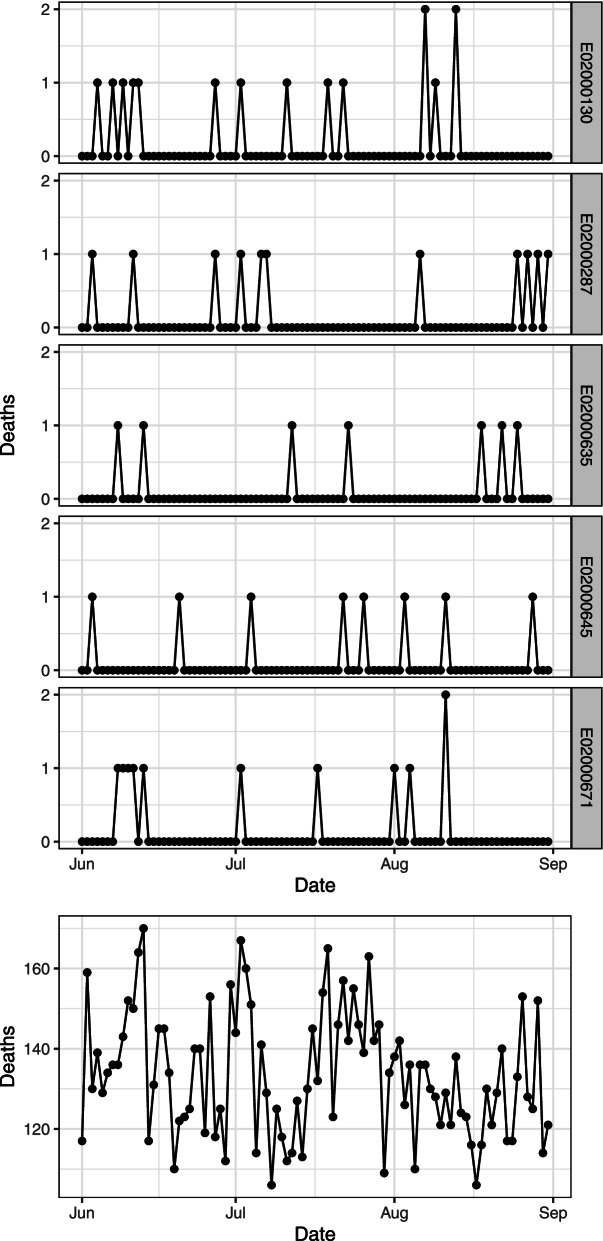
Daily series of deaths for all causes in the period June–August 2006 in five random MSOAs (top panel) and aggregated across all the 983 MSOAs of London (bottom panel)

The mortality series derived for five of the 983 MSOAs in the summer of 2006 are displayed in Fig. 1 (top panel). Each MSOA is characterised by no more than one or a few daily deaths, with most of the days totalling none. The data can be then aggregated further by summing across all MSOAs, thus defining a single daily mortality series for the whole area of London, shown in Fig. 1 (bottom panel). These fully aggregated data will be used later to compare the results of the CTS methodology with a traditional time series analysis.

The definition of the geographical units depends both on the research question and practical considerations. The areas should be representative of exposure and health risk processes, in addition to being consistent with the resolution of the available data. Choosing finely aggregated areas can better capture underlying associations in the presence of small-scale dependencies, but would pointlessly inflate the computational demand in the presence of low-resolution exposure data or risk mechanisms acting at wider spatial scales.

## Linking high-resolution exposure data

In this setting, one of the important advantages of the CTS design is the use of exposure measurements assigned to small areas (each of them representing a case), rather than averaging their values across large regions. The same applies to potential co-exposures or time-varying factors acting as confounders, that can be collected at the same small-area scale. Researchers have nowadays access to a variety of resources to retrieve high-resolution measurements of a multitude of risk factors across large populations. These resources include clinical and health databases, census and administrative data, consumer and marketing company data, and measurement networks, among others [3].

Environmental studies, for instance, can now rely on climate re-analysis and atmospheric emission-dispersion models that offer full coverage and high-resolution measures for a number of environmental stressors. In this case study, we extracted temperature data from the HadUK-Grid product developed by the Met Office [10]. This database includes daily values of minimum and maximum temperature on a 1 × 1 km grid across the United Kingdom. These data were averaged to derive mean daily temperature values and linked with the mortality series.

**Fig. 2:**
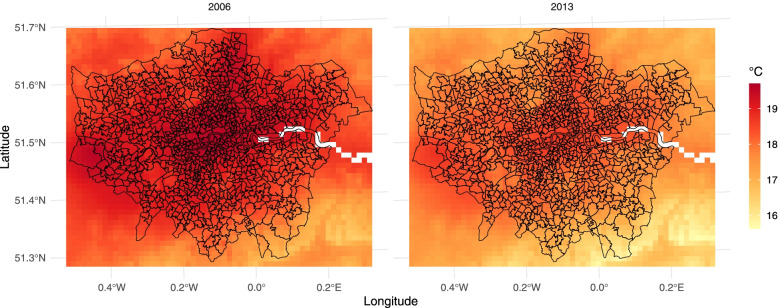
Average summer temperature (°C) in 2006 (left) and 2013 (right) in a 1 × 1 km grid of the London area, with superimposed the boundaries of the 983 MSOAs

The linkage process consists in spatially aligning the two sources of information, namely the polygons defining the 983 MSOAs and the intersecting grid cells with corresponding temperature data. Figure 2 displays the two spatial structures, with the average summer temperature in the two years in each of the grid cells overlayed by the MSOA boundaries. The maps show the spatial differences in temperature within the areas of London, with higher values in more densely urbanised zones.

**Fig. 3:**
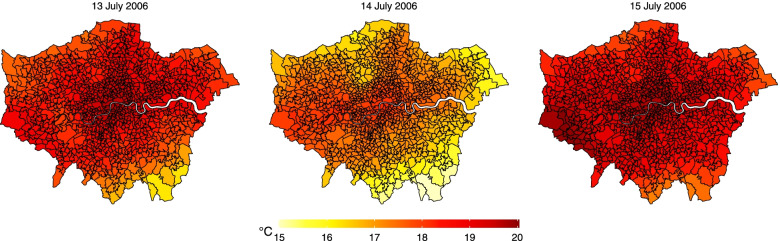
Mean temperature in three consecutive days (13–15 July 2006) across the 983 MSOAs of London

The alignment procedure is carried out using GIS techniques to compute the area-weighted average of the cells intersecting each MSOA, with weights proportional to the intersection areas. This step creates MSOA-specific daily series of temperatures that can be linked with the mortality data. The results are illustrated in Fig. 3, which show the temperature distribution in three consecutive days in July 2006, demonstrating the differential temporal changes of temperature across areas of the city. The same linkage process can be applied to other exposures or confounders, each potentially defined over different spatial boundaries.

An important advantage of the CTS design is the possibility to use data disaggregated at smaller scales, thus capturing differential changes in exposure across space and time, compared to traditional analyses using a single aggregated series that rely entirely on temporal contrasts. Even in the absence of measurement errors in both disaggregated and aggregated analysis, the former is therefore expected to result in more precise estimates. In this specific example, though, the gain in precision can be limited, as Fig. 3 indicates that the temporal variation seems to dominate compared to spatial differences. The two components of variation can be quantified by the average between-day and between-MSOA standard deviations in temperature, respectively. Results confirm the visual impression, with a temporal deviation of 3.0 °C compared to 0.4 °C of the spatial one.

## Main analysis

The CTS design allows the application of flexible modelling techniques developed for time series analysis, but without requiring the aggregation of the data in a single series. The modelling framework is based on regression models with the following general form:

1 $$g\lbrack\text{E}(y_{it})\rbrack=\xi_{i\left(k\right)}+f(x_{it},\ell)+\sum_{j=1}^js_j\left(t\right)+\sum_{p=1}^ph_p\left(z_{ipt}\right)$$

The model in Eq. 1 has a classical time series form, with outcomes ${y}_{it}$ collected along time $t$ modelled through multiple regression terms [11]. Specific functions can be used to define the association with the exposure of interest $x$, potentially including delayed effects through the inclusion of lagged values $x_{t-\ell}$ along lag period $\ell=0,\dots,L$. Other terms can be represented by functions modelling the underlying temporal trends using multiple transformations of $t$, and potential time-varying predictors $z$. The main difference from traditional time series models is in the presence of multiple series for cases represented by the index $i$. In particular, cases define matched *risk sets*, with intercepts ${\xi }_{i}$ expressing baseline risks varying across observational units. The risk sets can be stratified further by defining different intercepts ${\xi }_{i(k)}$ for each time stratum $k$, thus modelling within-case variations in risk. The regression is efficiently performed using *fixed-effects* estimators available for different outcome families [12, 13].

In our illustrative example, ${y}_{it}$ represents daily death counts for each of the $i=1,\dots ,983$ MSOAs. The risk association with temperature $x$ is modelled through a distributed lag non-linear model (DLNM) with a *cross-basis* term [14]. This bi-dimensional parametrisation is obtained using natural cubic splines defining the exposure–response (two knots at the 50^th^ and 90^th^ temperature percentiles) and lag-response (one knot at lag 1 over lag period 0–3) relationships. The other terms are two functions of time $t$, specifically natural cubic splines of day of the year with 3 degrees of freedom and an interaction with year indicators to model differential seasonal effects in 2006 and 2013, plus indicators for day of the week. Risk sets are defined by MSOA/year/month strata indicators ${\xi }_{i(k)}$, allowing within-MSOA variation in baseline risks in addition to common trends captured by the temporal terms in Eq. 1 above. The model is fitted using a fixed-effects regression model with a quasi-Poisson family to account for overdispersion.

**Fig. 4:**
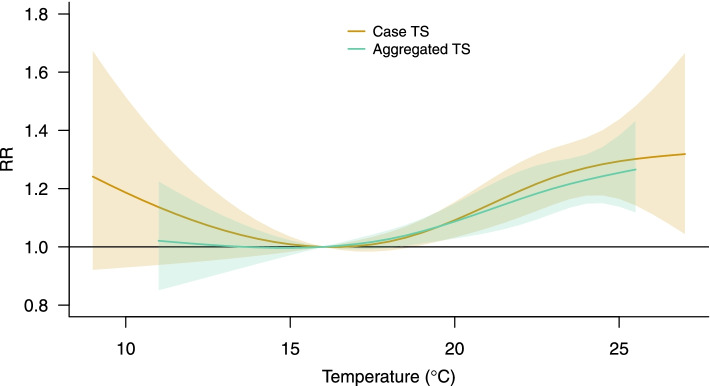
Exposure–response relationships representing the temperature-mortality risk cumulated within lag 0–3 estimated using the CTS model on data disaggregated by MSOAs (dark gold) and from the standard time series model with the aggregated data (green)

Results are displayed in Fig. 4, which shows the overall cumulative exposure–response curve (dark gold) expressing the temperature-mortality association. The curve indicates an increase in mortality risks above 16 $^\circ$ C, the optimal value corresponding minimum mortality temperature (MMT). The left tail of the curve suggests an increased risk also for relatively cold temperatures experienced during the summer period.

The CTS model can be compared to a standard time series analysis performed by aggregating the data in single mortality (Fig. 1, bottom panel) and temperature series, the latter obtained by averaging the daily values across MSOAs. The model is specified using the same terms and parameterisation as above. The estimated relationship is added to Fig. 4 (green curve). The aggregated analysis reports the association over a narrower range, as local extreme temperatures are averaged out (see Fig. 3), and indicates slightly lower risks, in particular failing to capture the residual cold effects. As anticipated, there seems to be little gain in statistical precision from the CTS model, given that in this example the temperature variation is mainly driven by day-to-day variation more than by spatial differences.

## Assessing differentials in vulnerability

The analysis can be extended by introducing additional terms in the model of Eq. 1, for instance to control for confounders or investigate effect modifications. Associations with time-varying factors can be specified in the usual way through main and interaction terms included directly in the model. In contrast, the conditional framework of fixed-effects regression removes effects associated with time-invariant factors, which are absorbed in the intercepts ${\xi }_{i(k)}$ [12]. This ensures that potential confounding from such terms is controlled for by design, but has the drawback that their main effects cannot be estimated. Still, interactions with time-invariant terms can be specified to model differential health risks across small areas. In our case study, we apply this method to investigate vulnerability to extreme temperature depending on socio-economic status, represented by the index of multiple deprivation (IMD).

As mentioned above, small-area studies can rely on information collected at different geographical levels, but this requires all the variables to be re-aligned over the same spatial structure, as shown for mortality and temperature above. In this example. IMD scores (defined from 0 as the most deprived to 1 as the least deprived) were originally collected at the smallest census level, the lower super-output areas (LSOAs). Therefore, this information is first re-aligned by averaging the values by MSOA.

**Fig. 5:**
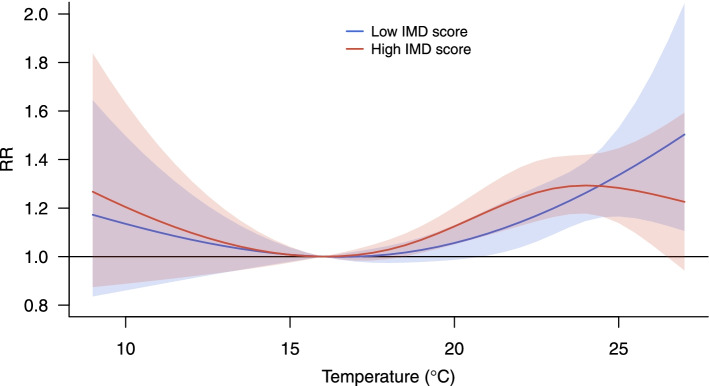
Exposure–response relationships representing the temperature-mortality risk cumulated within lag 0–3 predicted for less (blue) and more (red) deprived areas, defined by the inter-quartile range of the IMD score

The model is then extended by specifying a linear interaction between the cross-basis of temperature and the IMD score. The results are shown in Fig. 5, which displays the overall cumulative exposure–response curves predicted for low (in blue) and high (red) IMD scores, with values set at the inter-quartile range. The graph suggests little evidence of differential risks by deprivation, as confirmed by the likelihood ratio test (accounting for overdispersion) that returns a *p*-value of 0.73. It is worth noting, however, that this lack of evidence can be explained by the limited statistical power due to the short study period (two summers).

## Discussion

This contribution presents a tutorial on the extension of the CTS design for the analysis of small-area data. The tutorial illustrates the analytical steps using a real-data example, and it discusses practical issues, for instance linkage procedures and data analysis, as well as methodological aspects. The case study uses publicly available datasets with data and R code documented and made available in a GitHub repository. The example is therefore fully reproducible and can be easily adapted to other settings for epidemiological analyses using small-area data.

The main feature of the CTS design is the embedment of flexible time series methods within a self-matched framework based on multiple observational units. This setting offers strong control for both time-invariant and time-varying confounding as well as the possibility to model complex temporal relationships using finely disaggregated data. These aspects are demonstrated in the case study illustrated above. Specifically, the stratification of the baseline risk removes structural differences between MSOAs, while allowing control for area-specific temporal variations on top of common trends modelled through interactions between splines terms and year indicators. Likewise, the time series structure lends itself neatly to the application of distributed lag linear and non-linear models to define complex exposure-lag-response relationships. Finally, the design can improve the characterisation of the association of interest by providing both spatial and temporal contrasts. This is demonstrated in the case study example, where we show how the case time series framework can account for local exposure differences, for instance due to heat island effects, and allows investigating geographical variations in vulnerability.

The advantages of small-area studies, when compared to more traditional approaches based on largely aggregated data, are obvious. First, measurements of health outcomes and risk factors at a small scale are expected to represent more appropriately risk association mechanisms and to provide better control for confounding, thus reducing potential biases that affect ecological studies [7]. Even in the absence of classical measurement error, whereby the aggregated exposure value is a valid proxy of the true population average, small-area studies can reduce the Berkson-type error and therefore increase the statistical power [15]. As discussed in the example above, the gain in precision is proportional to the geographical differences in exposure across the study area relative to temporal variations.

The CTS design can be compared to other approaches previously used for epidemiological analyses using small-area data. Traditionally, spatial and spatio-temporal analyses are performed using Bayesian hierarchical models [6]. These methods provide a powerful framework that accounts for spatial correlations and allows geographically-varying risks, but they present high computational demands that pose limits in the analysis of large datasets and/or complex associations. In contrast, the CTS design offers a flexible and computationally efficient scheme to analyse temporal dependencies while removing entirely potential biases linked to between-area comparisons. As an alternative approach, other studies have replicated two-stage designs developed in multi-city investigations to small-area analyses [16, 17]. However, this method encounters estimation issues in the presence of sparse information due to finely disaggregated data, and for instance it would be unfeasible for the analysis of MSOAs in the illustrative example (see Fig. 1). Conversely, the CTS design sets no limit to data disaggregation, being applicable with the same structure to individual-level analyses. This aspect is shared by the case-crossover design, a popular methodology previously proposed in small-area analysis [18, 19]. In fact, the CTS methodology can replicate exactly the matching structure of the case-crossover scheme [20], while allowing a more flexible control for temporal trends and modelling of temporal relationships, as demonstrated in the illustrative case study.

Some limitations must be acknowledged. First, similarly to traditional time series methods, the CTS design is only applicable to study short-term risk associations with time-varying exposures, and cannot be used to assess long-term health effects. Likewise, its application in small-area studies is still based on aggregated data and it essentially retains an ecological nature. However, the extreme stratification can prevent some of the associated biases, and it is worth noting that the CTS methodology can be seamlessly applied to individual-level data, when these are available. Finally, its time series structure is ideal for modelling complex temporal dependencies and trends, but presents limitations in capturing spatially correlated and varying risks.

In conclusion, the CTS methodology represents a valuable analytical tool analysis of small-area data. The framework is highly adaptable to various data settings, and it offers flexible features for modelling complex temporal patterns while controlling for time-varying factors and trends. The availability of data collected at small-area level provides opportunities for its application in a variety of epidemiological investigations of risk associations.

## Data Availability

The data, software and code for replicating the analysis and complete set of results are made fully available in a GitHub repository (https://github.com/gasparrini/CTS-smallarea). The original data, at the time of writing, were publicly available from online resources. Specifically, the number of daily deaths by MSOAs of London in the summers of 2006 and 2013 was published by ONS (link); the geographical boundaries of the MSOAs and the lookup table between LSOAs and MSOAs (for the 2011 census) were available at the Open Geography Portal of ONS (link) and the data Open Data portal of GOV.UK (link); the gridded daily temperature data temperature data in the HadUK-Grid database from the Met Office were extracted from the Centre for Environmental Data Analysis (CEDA) archive (link); the IMD scores by LSOAs (for the year 2015) were provided at GOV.UK (link). Additional information on the linkage procedure with the original resources to obtain the final data, as well as the use of the R scripts, are provided in the GitHub repository.

## Abbreviations

CTS: Case time series
DLNM: Distributed lag non-linear model
MSOA: Middle layer super output area
LSOA: Lower layer super output area
IMD: Index of multiple deprivation
ONS: Office for National Statistics
